# The impact of welfare technology on care ethics: a qualitative analysis of healthcare professionals and managers’ experiences with welfare technologies

**DOI:** 10.1186/s12913-024-12187-2

**Published:** 2025-01-14

**Authors:** Brita Gjerstad, Ragnhild Gjerstad-Sørensen, Inger Lise Teig

**Affiliations:** 1 Department of Social Studies, University of Stavanger, Stavanger, Norway; 2 NORCE Norwegian Research Centre, Stavanger, Norway; 3 Department of Global Public Health and Primary Care, University of Bergen, Bergen, Norway

**Keywords:** Autonomy, Care ethics, Healthcare professionals, Qualitative research design, Welfare technology

## Abstract

**Background:**

Providing healthcare for the elderly population is challenging due to a shortage of staff. The challenge is addressed by increased use of technology. The article explores the impact of welfare technology on healthcare personnel’s care ethical considerations in Norway’s primary healthcare sector. Through a qualitative study of how healthcare professionals, managers, and technology suppliers understand and perceive welfare technology in healthcare, we examine whether instrumental values displace care-ethical values in primary healthcare practices.

**Methods:**

The study is based on a qualitative analysis of interviews with healthcare workers, healthcare managers and technology suppliers in primary healthcare in Norway. Semi-structured interviews were conducted with managers and quality developers at the municipal administrative level and healthcare managers and staff in healthcare units. Interviews with suppliers/manufacturers of welfare technology (GPS, pill dispensers and robotics) were also conducted. We combined an inductive approach with theoretical exploration as we alternated between the empirical data, a thematic approach, and theories of technology and care ethics.

**Results:**

In the analysis of the empirical material, we identified two overarching themes that were related to our research question: 1) demands and solutions and 2) two sides of autonomy. The informants generally highlighted the benefits of welfare technology, but the informants were also ambiguous about the use of welfare technology. Autonomy was seen as an important value but was attached with ambivalence.

**Conclusion:**

Care ethical considerations are significantly present within healthcare professionals’ understandings of technology even though managers and technology suppliers were advocating for welfare technology in a more instrumental sense. Despite extensive acclaim for user autonomy, healthcare personnel make decisions about care and technology use independently of the resource situation. They hold onto a professional room of freedom in between the patient’s needs, available resources, and suitable technology. They are sceptical about applying technological solutions if they suspect it will lead to potentially adverse consequences, such as loneliness or increased insecurity due to technological illiteracy. By engaging a relational autonomy approach in their care practices, healthcare professionals control technology rather than submit to technology and we see that rather than being displaced by technical-economic reasoning, care ethical reasoning also accommodates technology.

**Supplementary Information:**

The online version contains supplementary material available at 10.1186/s12913-024-12187-2.

## Background

Norway, like many other countries, faces challenges in providing healthcare for its elderly population due to a shortage of staff [1]. Currently, Norway is short of 4,650 nurses [2] and other healthcare personnel are in short supply. It is estimated that by 2040, the number of nurses needed in Norway will increase to 30,000 [3]. These challenges are addressed by increased use of technologies [4, 5]. In Scandinavia, technologies aimed at healthcare are often referred to as “welfare technology” [6, 7]. While there is no common definition [4, 6, 7], it is usually referred to as technology that in different ways supports people in their daily lives in their homes. In this study, we rely on a commonly used definition in Norway, which states that welfare technology is “Technology that can contribute to increased security, safety, social participation, mobility and physical and cultural activity, and that strengthens individuals’ ability to manage for themselves in everyday life despite illness and social, mental or physical disability. Welfare technology can also function as technological support for next of kin and otherwise help to improve accessibility, resource utilisation and the quality of service provision. Welfare technology solutions can in many cases prevent the need for services or hospitalisation” ([4], p. 2). Examples are medicine dispensers, fall alarms, and sensor-controlled curtains to mention a few. Norwegian governmental plans and strategies for strengthening primary healthcare emphasise welfare technologies which are accompanied by guidelines, recommendations, standardisations, evidence-based knowledge, and the National Welfare Technology program [8], all aimed at supporting the implementation of technology into the healthcare sector. The optimistic expectations of Norwegian national authorities regarding technology in healthcare services are striking [9–12], but this is not unique to Norway. The demographic trends are like those in the rest of the Western world, and many studies view technology as a solution to the growing demand for healthcare services [6, 7, 13, 14]. Lupton [15] refers to this belief in technology as “Techno-Utopia” or as an era characterised by attempts “[...] towards the digitalisation of as many elements of healthcare as possible” ([16] p. 707). Health policy discourses on technology often adapt an uncritically positive tone [16]. However, technology and care are frequently seen as belonging to separate ethical spheres. Technology is associated with values such as efficiency, speed, productivity, resource exploitation, simplification, and accuracy [17], while care is linked to compassion, competence, confidence, conscience, and commitment, within a framework of sharing and mutual respect [17–19]. While technologies advance rapidly, ethical considerations tend to evolve more slowly, shaped by ongoing processes of learning, failure, and reflection [20]. When new technologies are implemented, established ethical routines are often lacking [20], and their impact on care ethics remains underexplored [17, 21, 22]. However, we know that new technologies can introduce new ethical challenges [23] and reshape well-known dilemmas [22].

### Aim and research question

Our interest in this topic stems from the growing push for technology in the primary healthcare sector, driven largely by national authorities and technological suppliers who promote it as a solution to resource constraints. The aim of this study was to explore the impact of welfare technology on healthcare personnel’s care ethical considerations in Norway’s primary healthcare sector. The theoretical framework used in this study is twofold, centred on the research question of how increased use of technology may create tensions between instrumental values and care-ethical values. Through a qualitative study of how healthcare professionals, managers, and technology suppliers understand and perceive welfare technology in healthcare, we examine whether instrumental values displace care-ethical values in primary healthcare practices.

## Theoretical framework

### Technology as socially constructed

We apply a social constructional perspective on technology, implying that technology is never neutral. From this perspective, technology is designed according to designers’ values and images about what users want, do, wish for, and are capable of [24]. These values and imaginaries are inscribed into the technology’s functionality and material form. While such inscriptions influence how we understand and use technology, they do not fully define it, as technology is not limited to its inherent properties. Instead, it is interpreted in different ways depending on the user’s context. In this way, social and cultural factors play a significant role in shaping these interpretations which vary between various actors [25]. The concept of the “technological frame” helps define this context by illustrating how interactions are structured and how they shape actors’ thinking and acting [26]. Healthcare personnel, managers, clients, and designers operate within different technological frames, meaning that designers and managers cannot assume that technology will be understood or used exactly as intended. Through ongoing negotiations among these actors, one interpretation may eventually become dominant, gaining status as the correct understanding [25]. However, this does not imply unanimous agreement; individuals who hold differing views may find their perspectives marginalised as deviant. By focusing on interpretations, the social construction of technology acknowledges that implementing technology is a complex, unpredictable process. It challenges the notion of technological determinism [26], contrasting with the common assumption in literature and policy documents that there is a single developmental trajectory everyone will eventually follow [27]. According to this assumption, some individuals, sectors, regions, or countries may be further along the path, but they are all thought to be heading in the same direction.

While social factors shape how we understand technology, technology, in turn, influences how we think and act. New technologies offer choices that can impact how we evaluate options. In addition, it can be difficult to separate technological factors from social ones, as the two are closely intertwined. For example, in our study, distinguishing between a technology like a pill dispenser and the service it provides is challenging. Bijker’s concept of “the seamless web of technology and society” [28] captures this mutual influence, suggesting that it is not always clear whether a problem is technical or social in nature [26] - or, in the context of our study, ethical.

### Care ethics

Since the foundation of all care lies in the interdependence of people’s lives [29], theories that address this mutual dependency offer a valuable approach. Care ethics, which serves as the professional ethical framework for healthcare personnel, is a distinct ethical theory focused on relationships. That personnel are bound by professional ethics is not the same as saying that everything they do is in line with these ethics. Unlike other ethical frameworks, it cannot be reduced to abstract principles or rigid rules, as it focuses on the dynamic and relational aspects of care [29]. As Pettersen [29] emphasises, the caregiver’s obligations are shaped by their involvement with specific individuals. Rigid hierarchical rules alone cannot capture the complexity of nurse-patient relationships [30]. Care, therefore, can be understood as embodying values such as attentiveness, responsibility, competence, and reciprocity [31]. Care ethics reject the idea of human beings as independent entities, instead recognise dependency as a fundamental aspect of human life, especially within the context of care [31]. This relational approach views individuals through a lens that "[…] normalises dependency and vulnerability as basic features of the human condition” ([32] p. 102).

In society at large, as well as in health, independence is often upheld as a central value [33], and autonomy, closely tied to independence, remains a key ethical principle in medicine [34]. While care ethicists do not dismiss the value of autonomy, they challenge its libertarian interpretation [35], which equates maximum freedom of choice with minimum restrictions on individual choice. In this view, autonomy is understood as negative freedom - freedom from interference by others – based on cognitive decision-making abilities and the need for independence and freedom to make (rational) choices [36]. Care ethics, by contrast, propose an alternative concept known as “relational autonomy” [33, 35]. This perspective incorporates societal dimensions, understanding the patient as a relational being embedded within a broader context [35]. The caregiver’s approach is not solely focused on the patient’s cognitive abilities, but on the relationships and contexts that shape care, moving away from an emphasis on individual self-interest [33]. The relational emphasis in care ethics critiques the individualistic ideology that often portrays individual autonomy and freedom of choice as solutions to societal challenges, including those in healthcare [19].

## Methods

### The study context

In Norway, welfare services such as healthcare and elderly care are publicly funded through taxes and predominantly provided as public services. Local democracy is strong with responsibilities shared across different governmental levels. The central government holds overarching authority and supervises the municipalities, which, regardless of size, are the primary providers of welfare services. These services are equally accessible to all citizens [37]. Norwegian health policy strategies prioritise independence in daily life by increasing the use of welfare technology to “[…] strengthen the individual’s ability to manage themselves in everyday life despite illness and social, psychological or physical impairment” ([37], p. 99). The importance of autonomy using technology has been highlighted in both past [38] and forthcoming^1^ healthcare reforms. Norwegian citizens, in general, have a high level of digital competence compared to other countries, with access to ICT and the Internet among the highest in the world. However, senior citizens are less digitally engaged [39] and almost all non-users of technology belong to the elderly population [40]. In comparison to many other nations, Norway likely presents a more technology-friendly and technology-dependent context, with greater financial resources to support the implementation of new technologies. The Norwegian state has been proactive in digitalising its welfare system, eager to realise the potential benefits of technology [41].

### Design

This study qualitatively explores how healthcare professionals, healthcare managers, and technology suppliers in Norwegian primary care perceive and reflect on welfare technology, with a focus on potential tensions between instrumental and ethical values. Specifically, it examines their understandings of pill dispensers, localisation devices (GPS) and personal security alarms (fall alarms). The analysis follows Braun and Clarke’s [42, 43] guidelines for thematic analysis, aiming to uncover both the manifest and latent meanings of the informants’ experiences with these technologies. Recognising that tools, objects, facts, and technologies are always embedded in social practices, the study takes a contextual approach, acknowledging that technology derives its meaning from its specific use [44]. The epistemological assumption underpinning this study is social constructivism, which assumes that technology is socially shaped. As Bjiker states: “[t]echnologies do not merely assist in everyday lives, they are also powerful forces acting to reshape human activities and their meanings” ([28], p .66). With the exception of saturation, which is not required when using thematic analysis [45], the study adheres to the Consolidated Criteria for Reporting Qualitative Research 32‐item checklist to ensure transparency [46].

### Participants

As part of this study, we conducted nine interviews (six individual and three group interviews) with participants from municipalities and four interviews (three individual and one group interview) with welfare technology companies during the winter and spring of 2022. All participants were recruited based on direct contact with the municipalities and the technology companies. The municipalities were chosen based on online official information on the respective use of technology in the municipalities. The selected companies are central suppliers of welfare technology to Norwegian municipalities. All together the sample consisted of 17 participants in 13 interviews (described in Table 1). The municipal part of the study was conducted in three municipalities in Norway that varied geographically in size, location, and density, and participants were purposively sampled to include employees at a managerial (administrative and prime care unit level) and advisory staff level, who were responsible for introducing and providing welfare technology in daily practice. We identified relevant managers and advisors from information on the municipalities’ websites. We invited them to participate via email and attached an information sheet. On our request, the managers suggested further informants at lower levels in the municipality. To managers on the prime care unit level, the job also involved patient contacts, as their position entailed both managerial and nursing tasks. Municipality A is a northern rural municipality with long distances and a less populated structure. We conducted four individual interviews: two Heads of unit, one Advisor and one Deputy Manager. Municipality B is a rural, geographically expanded municipality in central/eastern Norway. We conducted one individual (High level Manager) and two group interviews (1. Two Team Leaders, 2. Head of Unit and Advisor) and Municipality C is a medium-sized city in central/northern Norway with a dense population structure. Here, we carried out one individual (High level Manager) and one group interview (two Mid-level Managers). We also conducted four interviews with the supply side of welfare technology: a GPS company (one individual interview, business manager), two pill dispenser companies (two individual interviews, one consultant and one general manager), and a robotics developer company (group interview with two developers). The participants gave their consent via email and/or orally at the beginning of the interview where we repeated the previously shared written information about anonymity (ensuring that no participants would be identifiable), confidentiality (indicating that only the researchers involved would have access to the data), the right to withdraw, and other relevant details. None of the participants declined.

**Table 1:** Participants

**Company/Municipality**	**Participant/Role**	**No. of participants**	**Method**
GPS company	Business Manager	1	Individual interview
Pill dispenser company A	General Manager	1	Individual interview
Pill dispenser company B	Consultant	1	Individual interview
Robotics developer company	Developers	2	Group Interview
Municipality A	Head of Unit A	1	Individual interview
	Head of Unit B	1	Individual interview
	Advisor	1	Individual interview
	Deputy Manager	1	Individual interview
Municipality B	High level Manager	1	Individual interview
	Team Leaders	2	Group Interview
	Head of Unit	1	Group Interview
	Advisor	1	
Municipality C	High level Manager	1	Individual interview
	Mid-level Managers	2	Group Interview
Total		17	13

### Data collection

The interviews were semi-structured with open-ended questions to allow the informants to emphasise and elaborate on different topics they were engaged in concerning the use of welfare technology. We had one interview guide for the interviews with informants from the municipalities and another guide for interviews with suppliers. The interview guides consisted of various questions aiming to uncover the participants’ descriptions and perceptions about the following topics: 1) the services and the products, 2) the use of technology (pill dispensers and personal security alarm), 3) perspectives on care and 4) the driving forces, advantages/disadvantages, ethical dilemmas, and possible challenged values (in care/care ethics) related to the use of care technology. The interviews were conducted through video calls and recorded after oral informed consent. The interviews lasted approximately one hour each and they were transcribed verbatim. The interviews were conducted by one of the authors, while the others were present during the interviews and followed up with questions at the end of the interview.

### Data analysis and ethics

Data was analysed using thematic analysis (TA) [42]. The six steps of TA do not indicate a strictly linear process as the researchers go back and forth between them [47]. All authors read the transcribed interviews, and categories and key themes were identified inductively through several rounds of individual readings and group discussions. The identified themes were then analysed and discussed collaboratively among the authors. We combined an inductive approach with theoretical exploration, moving between the empirical data, emerging themes and categories, and relevant theories of technology and care ethics. Although the analysis was largely data-driven, the theoretical framework also played an integral role. In discussions of care and care ethics, both theoretical and empirical, autonomy is almost an inevitable topic. Our theoretical framework highlighted the importance of autonomy, but the data allowed for a deeper exploration of its meaning and significance. The analysis ultimately led to two overarching themes, which can be described as "domain summary" themes—those organised around a shared topic ([47], p. 592)—while the sub-themes focus on shared meaning. The study was approved by the Office of Research Ethics (NSD) (id 982327).

## Findings

In the analysis of the empirical material, we identified two overarching themes that were linked to our research question: 1) demands and solutions and 2) two sides of autonomy.

### Theme 1: Demands and solutions

Demands and solutions are an overarching theme that encompasses several aspects. It consists of three subthemes.

#### Greater demands

All informants described an increased demand for municipal care services, driven by a growing number of elderly citizens with more severe health conditions. They noted that recipients of public services are in poorer health compared to previous years. One informant explained that current homecare services require more advanced care than before, as patients are referred to hospitals later and are discharged in a more vulnerable state. The overall picture presented by the informants is one of a significantly more challenging situation, with homecare services facing a sharp increase in responsibilities and tasks. This shift—from less vulnerable to more fragile patients, from lower to higher expectations, and from basic to advanced care—represents a profound transformation in municipal care services.

To some degree, family members take part in this transformation. One informant described her experiences with family members who, very soon after homecare is approved, expect that the care and the entire responsibility are passed on to the municipality. Several of the informants also found the patients much more demanding than before and that relatives and the patient herself are more aware of patients’ rights, have higher expectations, and are more likely to expect the health sector to meet patients’ social needs in addition to medical and caring needs. However, technology is not always the solution family members ask for. Some of the informants have experienced that relatives think that technology is at odds with good care, believing that healthcare personnel’s physical presence provides the best care:

“If we suggest technology, the response from relatives is often: No, they should rather have a visit. And you have to go and see [the patient], just go and check on them”. (Head of Unit A, municipality A).

According to the informant, relatives prefer physical presence. Relatives do not formulate the added value of visits compared to using technology in exact terms. However, they seem to want care that includes more than purely medical aspects. Alternatively, they do not trust the technology. Healthcare personnel, on the other hand, think that technology can work as an alternative to physical presence.

#### A need for action

Informants, including personnel who worked with clients, described that due to the lack of resources they “have to do something” to handle all the patients and to help everyone who needs care. Technology is a part of this “something”. One municipal manager expressed that healthcare workers think they “have no choice” and “no alternatives”, while other managers argued that they “cannot not” use welfare technology as it is unavoidable in the sense of being a safe and labour-efficient way of offering services. In addition, many informants described welfare technology as a good way of improving the quality of the services regardless of whether it saves costs:

“The gains are not necessarily limited to reduced costs, that is, saving money, it is also quality. When it comes to pill dispensers and medication, I think it is a quality for the patient to get medicine at the right time and feel that he/she can cope.” (High level Manager, municipality B).

“Other examples of quality improvements are when technologies can be used to avoid disturbances, for example by replacing nocturnal visits where there is a risk of waking the patient” (Advisor, municipality A).

A gain in the form of increased quality made managers positive about technology, but they found financial gains more difficult to specify and realise:

“And then you think that technology should solve and help us with scarce resources - to solve some tasks. But it is also difficult to realise that advantage and think you should save or cut employees. We don’t necessarily do that” (Deputy manager, municipality A)

The sense that technology is essential because of the demanding situation and its potential to improve the quality of services leads managers to appreciate technology even if the concrete gains are difficult to determine.

#### Formal implementation and practice

Most managers from the administrative level of the municipalities embraced welfare technology to a large degree and they did not question the use of technology in general. Technologies like pill dispensers and personal security alarms were seen as a highly needed and necessary part of the services, and unit managers described that they continually worked to implement technology in their units. All three municipalities explicitly promoted the use of welfare technology in their strategy plans and the benefits of welfare technology were broadly shared among the municipal managers. Informants from the healthcare units were informed of the welfare technology strategies of their municipalities and acknowledged that technology is an important part of providing homecare services.

However, technology suppliers expressed that welfare technology is not helpful for everyone and that limitations exist. One of them exemplified that pill dispensers may be wrong for patients with severe mental health problems. Other reservations about technology use also emerged. Informants with daily contact with patients carefully stressed that they approached each patient individually and applied technology only when useful and adequate to the patient’s condition and needs. They emphasised the need for close individual follow-ups and considerations of whether the patient is confident and feels safe with the technology. They were afraid of standardising care through technology, at the expense of individual needs. In addition, the need for technology can also quickly change due to changes in the patient’s condition, and technology decisions must therefore be evaluated continuously, according to the informants:

“It is re-considered and evaluated again and again. The patients who get pill dispensers – it is evaluated: Is it still justifiable and okay that they have this dispenser, or should we provide the care differently?” (Mid-level manager A, municipality C).

The undeniable benefits of welfare technology were also modified by the healthcare managers who highlighted the importance of individual adjustments:

“It’s all about the mapping, to map in advance – what’s right for you? Does the patient need social contact? Or can technology help?” (Deputy manager, municipality A).

Whether to use welfare technology for the specific patient is a shared decision between healthcare personnel, family members, the GP, and the patient herself. In specific cases, technologically skilled personnel are involved, but the patient always has the final word:

“But I think it is the user who should be in this - who is the one who decides for himself whether to use this or not. It is not the municipality who decides» (Mid-level manager A, municipality C).

According to the informants, patients are never forced to use welfare technology and can discontinue its use at any time. However, patients are informed about available alternatives, and healthcare personnel may sometimes attempt to persuade them of the technology’s benefits. They have observed that when patients are informed and feel confident in how the technology functions, they are more likely to adopt it.

### Theme two: two sides of autonomy

The benefits of welfare technology were highlighted by the informants. However, as the previous section conveyed, informants were also ambiguous about the use of welfare technology.

#### Patient’s autonomy benefits the municipality

Autonomy was highlighted as an important theme and value, but the material shows that informants also expressed an ambivalence related to autonomy. The strategy for welfare technology of one of the municipalities (B) states that welfare technology should ensure that citizens will be able to stay at home as long as possible, and many agreed, as one of the healthcare workers commented:

“The goal is for as many people as possible to be able to take care of themselves at home for as long as possible. This is the overall goal for this municipality. We are getting much older than before, and the life expectancy is very high here (…) If we are going to provide for everyone, this is what we must do [to use welfare technology] to get enough people to work and provide the service we are going to do”. (Team leader A, municipality B)

The opportunity to take care of yourself through welfare technology, as stated by the team manager above, was emphasised in the interviews. This autonomy appears as crucial for the municipalities to fulfil expectations and requirements for the services. One manager outlined the benefits of patients self-administering their medication with the help of technology:

We see the benefit in terms of them [the patients] getting the medicine at the right time. We don’t necessarily have to be there when they take the medicine. We don’t always need to be there to give them the medicine, they can take it themselves. Then we can come later in the day with the supervision we are supposed to have. We manage our time a little better. Actually, we have very good experience with it. (Head of Unit A, municipality A)

The view that autonomous patients are good for the municipality, is shared by technology suppliers who point out that the use of technology can provide flexibility and the possibility to re-organise services in more efficient ways.

#### Autonomy is an essential value

Health professionals viewed autonomy as even more important for the patients themselves. They believed that welfare technology empowers elderly individuals by promoting greater independence, enabling them to manage their own lives, bodies, and time with more control.

“We have many examples of patients in home care who sat and looked at the clock and wondered when they were going to get their morning medicine, who can now report that after they have received pill dispensers they can come and go from their own home when it suits them. . . We enable them to be independent”. (Head of unit A, municipality B)

Other professionals strongly stressed that welfare technology allows patients to exercise greater freedom, allowing them to choose whether or not to receive visits from healthcare personnel, ultimately fostering a sense of independence. Among the suppliers, this freedom was explicitly emphasised, as they viewed visits as a constraint. One of the suppliers described this limitation in the following way:

"Today, there are very many people who are deprived of the opportunity to live a more independent life. They are almost imprisoned in their own home. Because they will have visits from the home care services 2–3 times per day, only to give one dose of medicine in their hand." (Pill dispenser company A)

Visits can sometimes be disruptive, and healthcare personnel described that some patients prefer not to be checked, especially if they share a room with another person. Digital monitoring systems prevent disturbances during the night, and devices like pill dispensers, GPSs, and alarms reduce unwanted contact while maintaining safety. By reducing physical and social interaction, welfare technology also alleviated patients’ feeling of being a burden. Relatives were similarly affected by the increased use of such technologies. Many healthcare informants had experienced that devices like security alarms and pill dispensers eased the daily caregiving responsibilities and the anxieties of relatives. Families felt more reassured and relied more on the services provided, transferring more of the responsibility to the healthcare system. The technology suppliers explicitly highlighted autonomy as one of the most important aspects in designing and developing their welfare technology products. They wanted to offer a technology that enable patients to maintain independence despite the growing demand for home-based care. As two suppliers explained, autonomy was central to them because:

“It is deep in people, it seems, to be able to be independent, to fend for themselves and not be dependent on others.” (Pill dispenser company A).

“To handle one’s own medication is extremely important. Independence, coping, flexibility, not the least in everyday life, is of value for clients who depend on a service and who have to wait for a service provider to come.” (Robotics developer)

As the quotes illustrate, the informants use emphatic language to emphasise the significance of autonomy, clearly presenting it as a fundamental value.

#### Potential downsides of autonomy

While autonomy was applauded among all informants, healthcare personnel also reflected critically on the potential negative aspects of technology. One of the healthcare managers questioned whether the patients are becoming more independent and said:

“We must be aware of what we are doing”. (Head og Unit A, municipality A)

This manager pointed to the dilemma expressed by many other informants; that more autonomy for the patient requires closer follow-up to ensure that the patient’s condition is adequately monitored and to be able to detect and act on the deterioration of the patient’s condition. Increased independence for the patient was seen as a challenge as healthcare personnel ended up having less direct contact and had to largely rely on technology. Some of the healthcare personnel felt unsure in this situation. Furthermore, another tension with welfare technology that healthcare personnel expressed was whether more autonomy in healthcare services could safeguard a dignified life for the elderly.

“It is - I think it is - good care is linked to dignity in your life. That you feel that it is a dignified life at home. We are very keen to offer services that enable everyone to stay at home for as long as possible. But does it feel like a worthy life? Are you able to take care of yourself or not? I think it has to do with dignity”. (Head of Unit A, Municipality A)

As the informant questioned above, a dignified life was not seen as the same as an independent life. Many informants were concerned that the elderly were becoming lonelier with more technology and were unable to manage social aspects of their lives.

## Discussion

With the perspectives of ethics of care and technology as socially constructed, we examined the understanding of welfare technology among providers in the primary healthcare service in Norway. Our main findings revealed that despite the optimism and enthusiasm associated with it, the use of welfare technology brought tensions and ambivalence. All participants perceived the use of welfare technology as necessary and beneficial for the services as such, but healthcare professionals were more ambivalent and described a way of working that seems to make space for care ethical reasoning beyond using technology as a standard for all patients. Welfare technology was seen as an important means to solve the problem of personnel shortages they faced. By adding welfare technology and technically trained personnel to the services, managers had experienced, and they expected, that they could deliver services that were more organisationally and economically efficient. In addition, participants recognised that welfare technology helped patients become more independent from healthcare services provided by personnel through technological devices that allow them to manage and organise their lives more flexibly and autonomously. They perceived that welfare technology gives patients increased autonomy through greater freedom of choice and less control and restrictions in everyday life. The participants’ perspectives on welfare technology seem to merge technology and healthcare in a way that technology *becomes* healthcare.

The embrace of welfare technology as the main solution to the challenges in the services appears to be a kind of technical-economic reasoning with a focus on gains and efficiency that differs from care ethical thinking. Nevertheless, technology is never neutral but is interpreted in different ways according to the context [24, 25], and we find that participants’ understanding of welfare technology is shifting among the participants. Like managers, healthcare personnel found the use of technology imperative due to the emphasis on welfare technology to increase economic efficiency and the patient’s autonomy. However, they found room to choose not to use technology and thus to use their professional judgment concerning the patients’ individual needs. They involved different actors in their decisions about the pill dispensers, GPSs, and alarms, such as municipal personnel, general practitioners, next of kin and the patient herself. These decisions are not based on external rules or established principles (e.g., guidelines for applying welfare technology) but are grounded in ethical care assessments. This becomes evident in their use of a leeway that exists between the patient’s needs, available resources, and technology. Based on care ethics, and thus with a holistic perspective, their discretionary assessments go beyond the practical advantages, for example not being restrained by personnel visits. Their use of discretion in their care work shows that technology is not perceived as forced upon them, or mandatory, but rather to be applied according to what is required in the specific context. Technology is thus integrated into care practices without replacing care. To use Bijker’s [25]) concept, technology and care are woven together into a seamless web.

However, tensions between care and technological reasonings arise when participants discuss autonomy. Autonomy is often referred to by perceptions like “freedom”, “independence”, or “more self-control” and is described as an important quality of technology. However, their understanding of autonomy appeared more ambiguous than we expected. When suppliers emphasise that welfare technology enables patients to be freer in solving their practical needs and more independent from healthcare providers, technology becomes a tool for “managing on their own” – to choose for themselves and to make their own decisions. Health managers see that pill dispensers, alarms, and GPSs for elderly patients gave patients more chances to leave their homes for social encounters and pleasure and they became more independent of healthcare services. Patients also avoided unpredictable visits with pill distributions due to delays or unexpected events and disturbing night checks. When autonomy is framed as the freedom to make one’s own choices or as freedom from restrictions, it often emphasises independence *from* healthcare services, reinforcing the value of self-sufficiency. This interpretation of autonomy can be viewed as reflecting instrumental rather than care-ethical values. Autonomy as a central concept value has been explored in various studies [23, 48, 49], with traditional understandings emphasising the role of rational thought and decision-making [33]. Thus, the prominence of autonomy as a key theme in this study was not unexpected. However, the concept of autonomy emerged with some ambiguity. Firstly, the opportunity for the elderly to organise their life more independently and manage better on their own as both managers and suppliers claimed and argued for, was questioned by the health professionals. They had experienced that the elderly could not take full advantage of the autonomy the technology provided. This suggests that to the clients, the realisation of autonomy requires more than technology. Many elderly people suffer from reduced health and/or mobility problems, others have no family or few friends, and some lack competence in technology. In contrast to Sharkey and Sharkey [23], who highlight numerous benefits of robotic assistive technology for the elderly, our study shows that healthcare professionals are ambiguous regarding whether welfare technology genuinely enhances patients’ independence and autonomy. While such technology can empower patients to manage their daily lives more independently, the informants raised concerns about its social implications. Specifically, welfare technology does not consistently increase the elderly’s social contact or mobility; in some cases, it may exacerbate feelings of loneliness. Given that loneliness is already a prevalent issue among the elderly [50–52], the autonomy facilitated by welfare technology can inadvertently expose the lack of relationships, interests, or social connections in their lives and intensify the sense of loneliness and isolation. However, individuals may still feel autonomous and value this sense of autonomy, even if it does not align with their actual level of independence [49]. The ambivalence of autonomy that emerges in our study points to the argument that a narrow understanding of autonomy ignores our dependence on relationships, and that dependence is an essential feature of human life and should not be denied [32]. According to Davy [32], agency and autonomy are inherently relational, emerging through the support and empowerment provided by others. This perspective suggests that welfare technology should not only aim to promote independence but also consider the relational and social dimensions of autonomy.

Secondly, according to suppliers’ and managers’ view of welfare technology, the patient is given the ability and freedom to govern his/her own life and make his/her own choices independently of relationships with others, in particular healthcare professionals. This emphasis on welfare technology as the guarantee for autonomy is in line with the liberal understanding of autonomy that gives “[…] the capacity for self-determination and self-government, the feature of human subjects that grounds individual rights and prevents paternalism” ([32], p.104). However, when patients meet technology, they may be threatened by a lack of technological knowledge and insecurity which impair their capacity to make decisions and do not give them the freedom to self-govern or make their own autonomous decisions. The ability to secure individual autonomy through welfare technology in the sense of governing their own life independently of healthcare professionals' help and follow-ups is a different kind of autonomy than relational autonomy that occurs within and as a result of relationships and interactions [23, 53]. Therefore, autonomy as one of the central themes that emerges in our study is more ambivalent than expected as it is attributed to different values ​​and not a shared concept.

### Strengths and limitations

One limitation of this study is its exclusive focus on the provider side of health services as neither patients nor relatives were included. This focus was intentional, as there are few studies examining care ethical reasonings among healthcare managers and welfare technology suppliers. The selection process, which relies on managers suggesting more participants, has a strength in that it ensures relevant participants, but a weakness is that we do not know whether they were suggested based on attitudes (although the data give no reason to believe so as informants were both negative and positive).

A key strength of the study lies in our exploration of different perspectives on technology from healthcare managers and professionals in the public sector, alongside suppliers in private companies. This revealed an ambivalence surrounding autonomy. We believe our findings contribute valuable insights into how tensions and ambiguities take shape in these two interconnected yet structurally distinct areas of welfare provision. Although we did not investigate the demand side of healthcare, we suggest that the views and perspectives of welfare technology from providers may influence patient expectations and experiences. Future research should explore this area further.

## Conclusion

This study has explored whether technical-economic values displaces care ethical values in primary healthcare. The study provides insights into how technology is perceived within primary healthcare settings in Norway. While managers and technology suppliers advocate for welfare technology in a more instrumental sense – viewing it as a solution to resources shortages, economic inefficiencies, and a means to enhance individual freedom of choice – care ethical considerations remain deeply embedded in healthcare professionals’ understanding of technology.

The study also reveals that despite the extensive emphasis on user autonomy, healthcare personnel carefully take many considerations into account when caring for patients. While the adoption of welfare technology may seem inevitable due to resource constraints, healthcare personnel make decisions about care and technology use independently of these limitations. They maintain a professional space of autonomy, balancing patient needs, available resources, and appropriate technology. Despite the pressure to adopt technological solutions in response to challenging circumstances, healthcare personnel remain cautious, particularly when they suspect that technology might lead to unintended consequences such as loneliness or increased insecurity due to technological illiteracy. By embracing a relational approach to autonomy, healthcare professionals manage technology as a tool for enhancing care, rather than dictating care practices. Their decisions about whether to use technology are not confined to standardised, predefined procedures. Instead, these decisions are part of a deeper, more nuanced process where technology is thoughtfully integrated into care. Rather than being overridden by technical-economic values, care ethical reasoning has been expanded to thoughtfully incorporate technology.

## Supplementary Information

Supplementary Material 1. 

Supplementary Material 2. 

## Data Availability

The data will be available as anonymized transcripts from the corresponding author on reasonable request.

## Footnotes

1 “https://www.regjeringen.no/no/tema/helse-og-omsorg/helse--og-omsorgstjenester-i-kommunene/innsikt/bo-trygt-hjemme-reformen/id2919447/
