# Supplementary material for: The impact of welfare technology on care ethics: a qualitative analysis of healthcare professionals and managers’ experiences with welfare technologies
Source: BMC Health Serv Res. 2025 Jan 14;25:73. doi: 10.1186/s12913-024-12187-2 (PMC11730466; doi:10.1186/s12913-024-12187-2)
Supplement: Supplementary file 1 — Supplementary Material 1. [file 12913_2024_12187_MOESM1_ESM.docx]

**Interview guide Managers and Professionals**

***General management***

- How would you characterise the organisation (values, services, aims)?
- What challenges does the sector face (strategic and financial)
- What impact do you think the increased use of technologies in care has on the sector?
- Is it economically profitable to use more technology? Where are the gains?
- What kind of organizational support do you have for investing in technology? (strategic plans, budgets, network)

***Technology***

- How and when did the need for technology emerge?
- Who is involved in technological decisions and what are the different actors’ roles? (encourage, restrict, teach, maintain)
- What is emphasised in choosing technology? (practical, economic, ethical, safety)
- What are the criteria for success/failure?
- What are the presumed and actual consequences of the services - short and long terms? (For users, relatives, personnel, management, community
- Are there economic gains and efficiency of work, in your opinion?
- How is technology financed? (investment, operation, training, support)

***Users***

- How are end-users involved or not involved? If not, why?
- How are users seen/defined? (skills, needs, wishes, engagement)
- What is required from users to handle the technological devices?

***Perspectives on care***

- How do you think good care should be practised and organised?
- What place does technology have in care?
- Do you think certain values are strengthened or weakened by technology? (dignity, autonomy), if so, which and how?
- Do you think technology affects professionals in certain ways? How?
- Do you think that there are alternatives to technology (the technology you are using? If so, which and why?
- Do you think technology is useful for care, if so, how?
