# Supplementary material for: The impact of welfare technology on care ethics: a qualitative analysis of healthcare professionals and managers’ experiences with welfare technologies
Source: BMC Health Serv Res. 2025 Jan 14;25:73. doi: 10.1186/s12913-024-12187-2 (PMC11730466; doi:10.1186/s12913-024-12187-2)
Supplement: Supplementary file 2 — Supplementary Material 2. [file 12913_2024_12187_MOESM2_ESM.docx]

**Interview guide Suppliers**

***The technology provided***

- Can you describe the concrete product? (What is it, how it works?
- Why are you selling/delivering this specific product to this municipality?
  - Who was involved in this process, at what stage?
  - How did the collaboration with the municipality start, and who/what "controlled“ it? (tenders, innovation cooperation)
- What kind of needs would this technology provide? (municipal, staff, users, caregivers)
- Which needs do you think are most important?

***About users***

- Can you describe a typical user (also different kinds of users)
- What do they need to know? (Training, available information, skills)

***The production***

- How was the product developed and designed.
- What has been important in the development of this specific technology? (for users, professionals, municipality, community)
- How will the product contribute to solving customer/user challenges?
- What kind of business model do you use?? (Marked, competition, marked advantage)
  - How do municipalities pay you?
  - How can you make a profit?
  - What kind of competence do your company need?
- What are the expected consequences (of the specific technology)? (autonomy, responsibility, dignity, social life, safety, security)
  - For users
  - For professionals (relationships with service users, professionalism, attitudes to care)
  - For management
  - For relatives
  - For society
  - In the short and long term

***Perspectives on care***

- Are there any difficult ethical aspects (or tensions/dilemmas) with this technology?
- How do you think the healthcare services will improve when using your technology?
- What do you perceive as good service and good healthcare?
